# Supplementary material for: Reducing greenhouse gas emissions from pig slurry by acidification with organic and inorganic acids
Source: PLoS One. 2022 May 5;17(5):e0267693. doi: 10.1371/journal.pone.0267693 (PMC9070912; doi:10.1371/journal.pone.0267693)
Supplement: S1 Appendix — (DOCX) [file pone.0267693.s001.docx]

**S1 Appendix. Correlation between CH_4_ and pH**


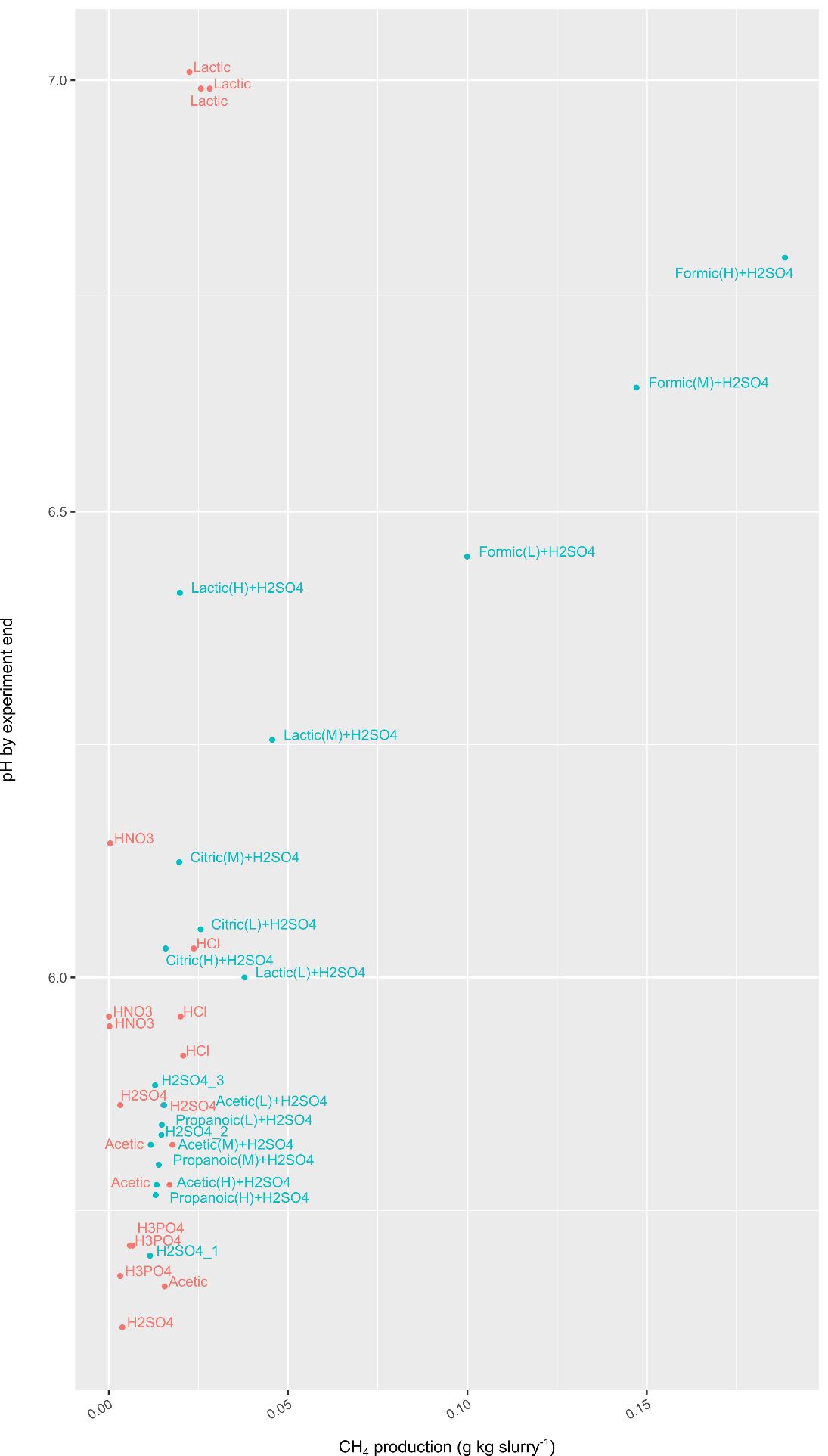


S1 Fig. Correlation between CH4 production and pH by experiment end in batch experiment A (18 days) and batch experiment C
