# Supplementary material for: Reducing greenhouse gas emissions from pig slurry by acidification with organic and inorganic acids
Source: PLoS One. 2022 May 5;17(5):e0267693. doi: 10.1371/journal.pone.0267693 (PMC9070912; doi:10.1371/journal.pone.0267693)
Supplement: S2 Appendix — (DOCX) [file pone.0267693.s002.docx]

S2 Appendix. NH_3_ emission rate


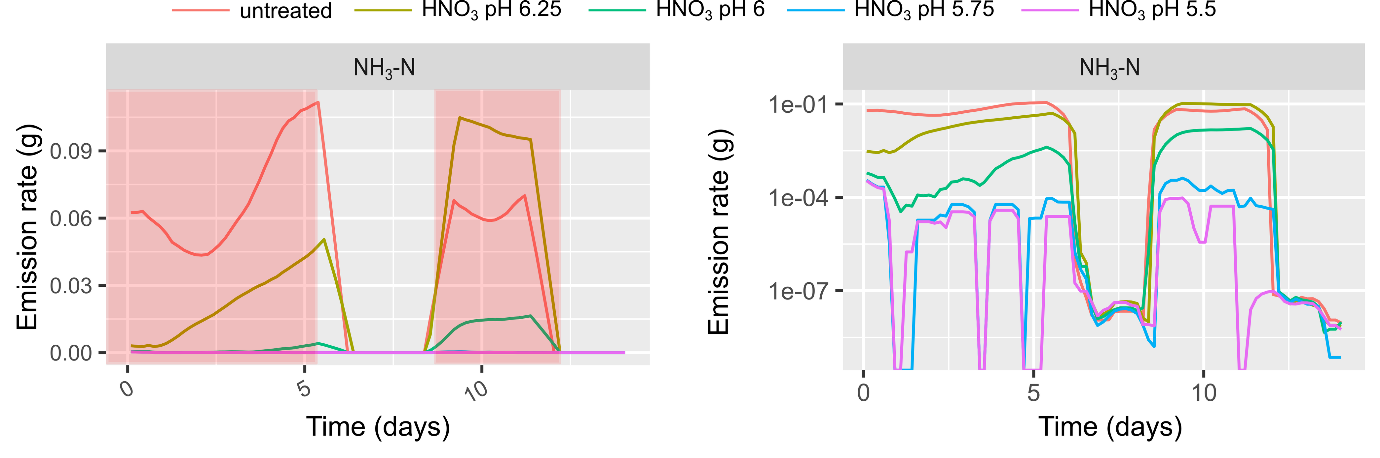


S2 Fig. Ammonia emission from continuous headspace experiment A (CHS A). To the left, with faded areas indicating when the nafion filter was not applied. To the right, log10 transformed y-axis.
