# Supplementary material for: Reducing greenhouse gas emissions from pig slurry by acidification with organic and inorganic acids
Source: PLoS One. 2022 May 5;17(5):e0267693. doi: 10.1371/journal.pone.0267693 (PMC9070912; doi:10.1371/journal.pone.0267693)
Supplement: S3 Appendix — (DOCX) [file pone.0267693.s003.docx]

**S3 Appendix.** log10 transformed emission rates


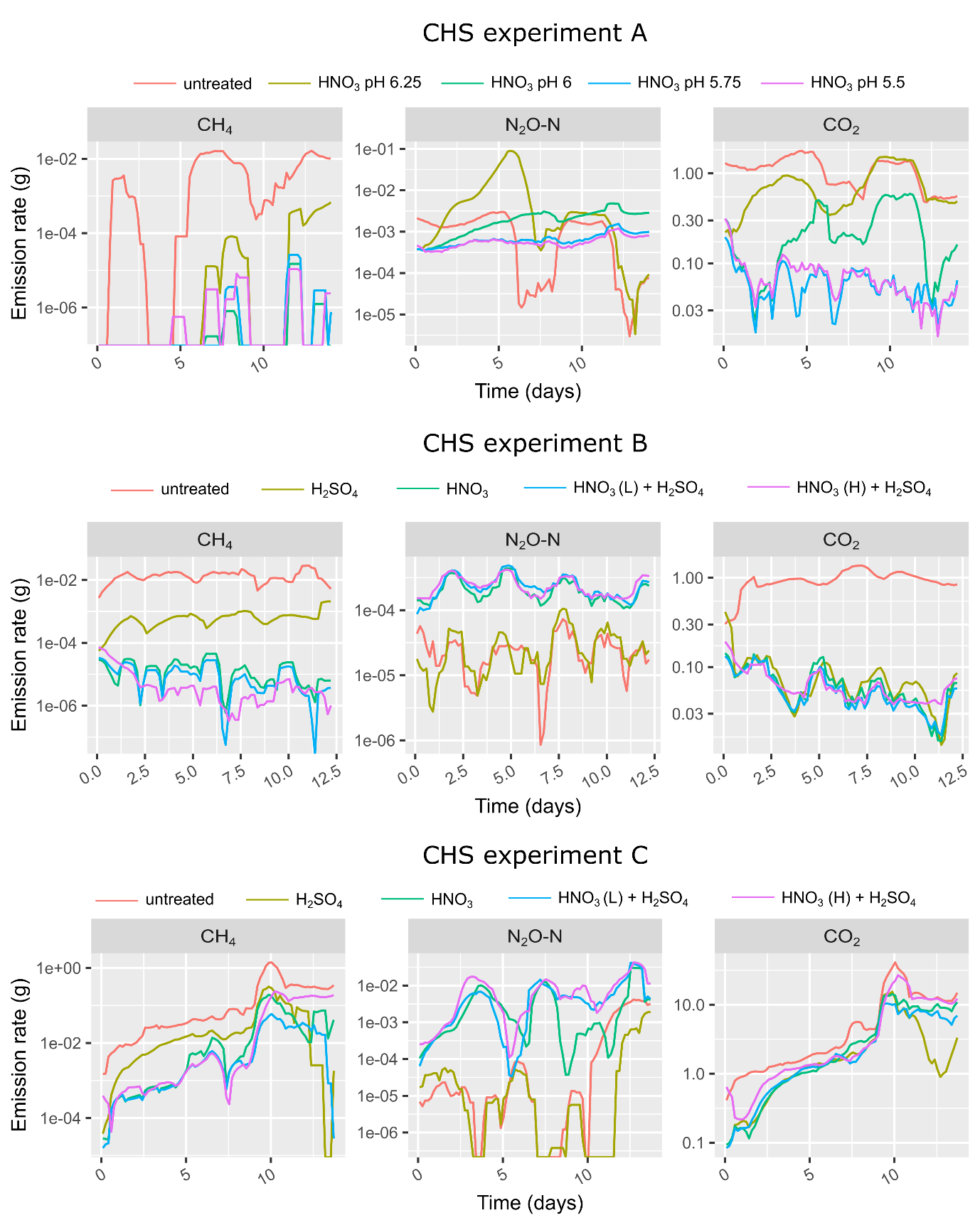


S3 Fig. Emission rates with log-transformed y-axis from CHS experiments A, B, and C
